# The Development of Videoconference-Based Support for People Living With Rare Dementias and Their Carers: Protocol for a 3-Phase Support Group Evaluation

**DOI:** 10.2196/35376

**Published:** 2022-07-20

**Authors:** Claire Waddington, Emma Harding, Emilie V Brotherhood, Ian Davies Abbott, Suzanne Barker, Paul M Camic, Victory Ezeofor, Hannah Gardner, Adetola Grillo, Chris Hardy, Zoe Hoare, Roberta McKee-Jackson, Kirsten Moore, Trish O’Hara, Jennifer Roberts, Samuel Rossi-Harries, Aida Suarez-Gonzalez, Mary Pat Sullivan, Rhiannon Tudor Edwards, Millie Van Der Byl Williams, Jill Walton, Alicia Willoughby, Gill Windle, Eira Winrow, Olivia Wood, Nikki Zimmermann, Sebastian J Crutch, Joshua Stott

**Affiliations:** 1 Dementia Research Centre Institute of Neurology University College London London United Kingdom; 2 Dementia Services Development Centre Bangor University Bangor United Kingdom; 3 Centre for Health Economics and Medicines Evaluation Bangor University Bangor United Kingdom; 4 School of Social Work Faculty of Education and Professional Studies Nipissing University North Bay, ON Canada; 5 School of Health Sciences Bangor University Bangor United Kingdom; 6 Division of Psychiatry University College London London United Kingdom; 7 Psychology and Language Sciences University College London London United Kingdom

**Keywords:** dementia, Alzheimer disease, frontotemporal dementia, posterior cortical atrophy, Lewy body dementia, Lewy body disease, primary progressive aphasia, young-onset dementia, early-onset dementia, atypical dementia, virtual, web-based, videoconference, videophone, support group

## Abstract

**Background:**

People living with rarer dementias face considerable difficulty accessing tailored information, advice, and peer and professional support. Web-based meeting platforms offer a critical opportunity to connect with others through shared lived experiences, even if they are geographically dispersed, particularly during the COVID-19 pandemic.

**Objective:**

We aim to develop facilitated videoconferencing support groups (VSGs) tailored to people living with or caring for someone with familial or sporadic frontotemporal dementia or young-onset Alzheimer disease, primary progressive aphasia, posterior cortical atrophy, or Lewy body dementia. This paper describes the development, coproduction, field testing, and evaluation plan for these groups.

**Methods:**

We describe a 3-phase approach to development. First, information and knowledge were gathered as part of a coproduction process with members of the Rare Dementia Support service. This information, together with literature searches and consultation with experts by experience, clinicians, and academics, shaped the design of the VSGs and session themes. Second, field testing involved 154 Rare Dementia Support members (people living with dementia and carers) participating in 2 rounds of facilitated sessions across 7 themes (health and social care professionals, advance care planning, independence and identity, grief and loss, empowering your identity, couples, and hope and dementia). Third, a detailed evaluation plan for future rounds of VSGs was developed.

**Results:**

The development of the small groups program yielded content and structure for 9 themed VSGs (the 7 piloted themes plus a later stages program and creativity club for implementation in rounds 3 and beyond) to be delivered over 4 to 8 sessions. The evaluation plan incorporated a range of quantitative (attendance, demographics, and geography; pre-post well-being ratings and surveys; psycholinguistic analysis of conversation; facial emotion recognition; facilitator ratings; and economic analysis of program delivery) and qualitative (content and thematic analysis) approaches. Pilot data from round 2 groups on the pre-post 3-word surveys indicated an increase in the emotional valence of words selected after the sessions.

**Conclusions:**

The involvement of people with lived experience of a rare dementia was critical to the design, development, and delivery of the small virtual support group program, and evaluation of this program will yield convergent data about the impact of tailored support delivered to geographically dispersed communities. This is the first study to design and plan an evaluation of VSGs specifically for people affected by rare dementias, including both people living with a rare dementia and their carers, and the outcome of the evaluation will be hugely beneficial in shaping specific and targeted support, which is often lacking in this population.

**International Registered Report Identifier (IRRID):**

DERR1-10.2196/35376

## Introduction

### Background

Support groups for people caring for or living with dementia (collectively, people affected by dementia) may be characterized as peer support groups facilitated by individuals with lived experience of dementia, educational or psychotherapeutic groups facilitated by professionals, or a combination of these components [1]. Support groups for people affected by dementia have been shown to reduce depression and carer burden as well as improve self-esteem, well-being, and quality of life [2,3]. These benefits have been shown in both in-person and virtual support group contexts [4-8] with multicomponent groups, involving input from peers and professionals, and a focus on psychoeducational and emotional support alongside experience-led guidance being most effective [9].

These groups tend to focus on providing support for the most common forms of dementia such as typical Alzheimer disease and vascular dementia. Although living with or caring for someone with any form of dementia can be a very isolating and lonely experience [6,10], this is a particular concern for people affected by rarer forms of dementia [11-13]. Rare dementia is an umbrella term referring to atypical, inherited, and young-onset conditions, often characterized by progressive difficulties with cognitive symptoms other than memory [14,15]. As individuals diagnosed with rarer dementias tend to be younger than those diagnosed with typical Alzheimer disease and vascular dementia, they have additional concerns including work, mortgages, and young families [16-19]. Rarer dementias also vary with regard to symptom presentation and impact on caregivers [20-22]. In addition, given the wide geographical spread, there is often a lack of tailored and specific local support available to people affected by these dementias, which is exacerbated by the often long journey to receiving a diagnosis [23].

In March 2020, the United Kingdom entered a nationwide lockdown owing to the COVID-19 pandemic caused by SARS-CoV-2. The restrictions resulting from the COVID-19 pandemic lockdown led to an increase in loneliness and isolation for people affected by dementia [24-26] and severely affected those with rarer forms of dementia [27]. For example, individuals with behavioral variant frontotemporal dementia often experience behavioral disinhibition and compulsions, making it difficult to follow government guidelines on social distancing, whereas those with semantic dementia find it difficult to understand the restrictions in place because of difficulties with comprehension. Those diagnosed with posterior cortical atrophy often rely on touch to help with navigation because of difficulties with vision and spatial awareness, which increases the likelihood of spreading the virus [28]. In response to the pandemic, there was a rapid implementation of a number of telehealth and tele-support services [25,29,30]. These services increase accessibility for people with long-term health conditions and those in rural areas, who would usually have to travel long distances to access health and social care services [31]. Online support groups may also provide an additional benefit for people affected by rarer dementias, even as restrictions lift, as due to the typically younger age of onset, carers and those with the diagnosis may still be working, potentially alongside managing childcare needs, and may therefore benefit from the additional flexibility that these groups provide [8,18,19].

Videoconferencing support groups (VSGs) are a type of web-based support [5,8,32]. VSGs have been found to have similar treatment outcomes when compared with in-person groups [32] and have also been shown to improve dementia caregivers’ mental health outcomes [7], including a decrease in burden and an increase in perceived social support and positive perceptions of caregiving [33]. In addition, Banbury et al [4], who implemented a 6-session videoconferencing peer support group for isolated carers of people with dementia, found that some group participants were more comfortable with videoconferencing than in-person groups, as they were in their own homes during the meeting, which felt like a *safe space* to share.

There is a lack of research on the benefits of VSGs specifically for people with rare dementias. In one of the few studies conducted with caregivers of people living with frontotemporal dementia, O’Connell et al [8] found benefits of VSGs, particularly in terms of being with caregivers who were in a similar situation to themselves with regard to age, relationship with the person with dementia, and their spouse’s diagnosis. Importantly, this group did not take place in the individuals’ homes but required group members to travel to their local health center to access the group, and group members reported difficulties in social connectivity because of the small screen sizes. Further research is needed to develop virtual support groups that can meet the unique needs of this population in an effective and sustainable way.

### Objectives

Considering the barriers to support group access for people with rarer dementias and the additional need for support during the COVID-19 pandemic, we aimed to develop a series of facilitated VSGs tailored to the needs of people affected by rare dementias. Using the study by Hales and Fossey [34] as a guide, along with principles related to user-centered design [35], we describe the development, coproduction, field testing, and evaluation plan for these groups.

## Methods

### Phase 1

#### Information, Knowledge Gathering, and Coproduction

Coproduction is an iterative process of discussions with experts by experience, clinicians, and academics to develop VSGs within the context of Rare Dementia Support (RDS). RDS is an organization that supports people affected by posterior cortical atrophy, familial Alzheimer disease, familial frontotemporal dementia, frontotemporal dementia, primary progressive aphasia, young-onset Alzheimer disease, and dementia with Lewy bodies. Before this process, RDS involved large in-person support groups for each disease type; held 3 to 4 times per year in London; smaller regional support groups; as well as one-to-one information, guidance, and advice. The in-person support groups (n=40 to >120) included a mixture of professional and member talks, question and answer, and smaller breakout group sessions (approximately, n=20) covering a range of topics, including postdiagnostic support, communication strategies, legal matters, regional support, activities, and caring in the later stages. It had been a long-held service development plan for RDS to offer smaller group sessions in addition to the larger support group meetings, enabling RDS to address topics in further depth and in a more intimate setting than was possible within the larger support group context because of limited time and large-group size.

#### Consulting Academic Literature

The development of VSG topics continues to be informed by the literature, along with consultation with academic and clinical experts. The Mental Health America Support Group Facilitation Guide [36] was adapted and used as a framework to guide facilitators throughout the online group discussion process. Given the lack of research into support services for rarer dementias described earlier, we focused on young-onset dementia (YOD) for the literature search because of the higher prevalence of rarer dementias in this population [14,37]. A recent study found that one-third of individuals with YOD receive their diagnosis via the memory clinic, a quarter via neurological services, and less than a fifth via young-onset specialist services [38]. The follow-up support that these individuals receive is incredibly variable, with nearly a third of individuals diagnosed with YOD reporting that they do not have any routine follow-up appointments [38]. Therefore, it is important that those affected by these conditions are educated on how to access health and social care services that may be able to provide additional postdiagnostic and ongoing support, as they may not otherwise be linked with these services. It is also important that people diagnosed with YOD have their own dedicated space [39], where they can share openly with their peers. In addition, people affected by YOD frequently experience feelings of predeath grief, which is associated with perceived stress, depression, and carer burden [40-42]. Predeath grief can include feelings of loss resulting from ongoing changes in roles, relationships, and identities [43,44] and may be of particular concern for individuals affected by YOD because of changes in areas such as employment, finances, and child support [17].

#### Consulting With Experts by Experience

Building on the initial experience of RDS and understanding the literature, RDS staff had a number of conversations with people affected by rare dementias in the early stages of the national lockdown. These conversations increased the awareness of the lack of support services available and highlighted the need for a support group specifically for people living with a rare dementia. They also highlighted specific themes to be covered (eg, educating RDS members on advance care planning and the role of health and social care professionals), which was particularly important given the lack of literature on support needs related to rare dementias.

#### Consulting With Clinicians

The subsequent small-group discussion themes were developed based on these discussions and integrated with the views of RDS (consultant) neurologists (n=7) who had years of experience interacting with people affected by rare dementias both individually and at support group meetings.

#### Consulting With Academic Experts

**Figure 1:**
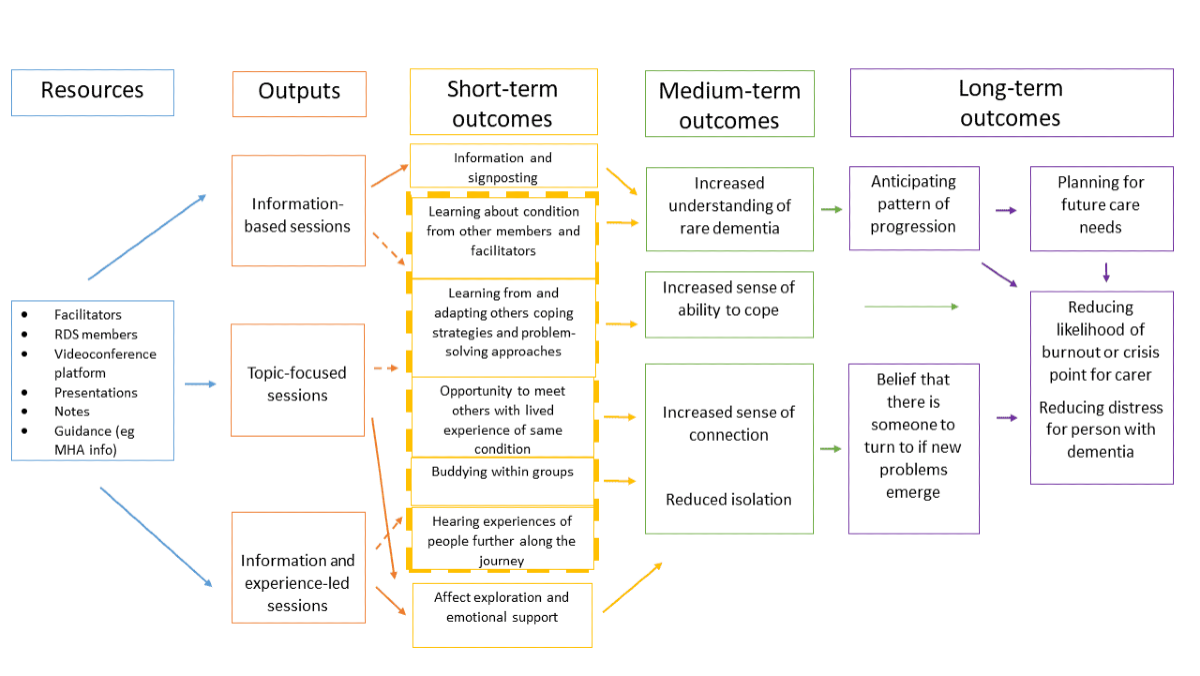
Output from phase 1 information and knowledge gathering. MHA: Mental Health America; RDS: Rare Dementia Support.

Clinical academics who worked on the RDS Impact Project [45] had a number of discussions to integrate theoretical perspectives with earlier discussions and consequent adaptations to the planned groups. These discussions with experts by experience, academics, and clinicians led to a decision on initial content and themes for the field-testing round of groups discussed below (Table 1), as well as a model describing flow through the groups and intended inputs and outputs (Figure 1).

**Table 1:** Videoconferencing support group (VSG) discussion content.

VSG discussion		Content
**Round 1**		
	Health and social care professionals	Neurology and memory assessment services Community support Postdiagnostic support Community mental health teams Support in the later stages
	Advance care planning	LPA^a^ Advance decisions and advance statements Registering as a carer Planning for hospital admission Care in the home and care homes Contingency planning Continuing health care Palliative care
	Independence and identity	Carer independence—challenges in maintaining interests and activities, and ways of managing this Maintaining independence and identity for people living with a rare dementia Carer identity—impact on sense of self, identification with the label of “carer”
	Grief and loss	Definition of grief and introduction to the concept of predeath grief Losses and feelings associated with grief Ambiguous loss Anticipatory grief Approaches to living with grief and loss Sharing ways members have adapted to grief Triggers for grief
	Empowering your identity	Health care professionals—who is involved in your care? Care planning—future planning, LPA, advance decisions and statements, role of general practitioner Independence and identity—adjusting to diagnosis, strategies for maintaining independence, and accepting help
**Round 2 (health and social care professionals and advance care planning groups were combined)**		
	Couples session	Independence: activities and interests, strategies to manage with difficulties Accessing support: navigating the health and social care maze, local support networks Planning together: advance care planning, choices and decision-making, emergency planning Living well
	Hope and dementia	What can challenge sense of hope when living with or supporting someone with a rare dementia Where hope can be found (including an object elicitation component) How hope changes over time
**Round 3**		
	Later stages program	This program will be open to those who are currently caring for someone in the later stages of dementia. Sessions will focus on sensory engagement, nutrition and swallowing, continuing health care and legal matters, care considerations, palliative care, pain management, and end of life.
	Creativity club	Intended for people living with dementia, the sessions will encourage members to share their ideas about painting, music, dance, cooking, and even gardening! Each session will include a short creative group activity, and attendees may be asked to bring examples to share or work on themes between sessions.

^a^LPA: Lasting Power of Attorney.

### Phase 2

#### Field Testing

The groups, based on the coproduced topics and process model detailed earlier, were subjected to field testing in 2 rounds between May 2020 and September 2020. The aims of field testing were to deliver a service during a pandemic while also making refinements to topics and understanding optimal processes for round 3 of groups where more formal evaluation is proposed.

#### Recruitment

RDS members (approximately, n=2000) received an email with the dates and a brief description of the VSG topics. They were asked to respond with their preferred groups as soon as possible, and recruitment for the groups was closed when the groups reached capacity (round 1=96 spaces available; round 2=132 spaces available).

#### Participants

In total, 154 RDS members (N=175; round 1: n=76, 43%; round 2: n=99, 57%) registered across the first 2 rounds of the VSGs, with 21 of those members registering for both rounds. These members included people living (n=27, 15%) or caring for someone now or in the past (n=127, 73%) with a diagnosis of a rare dementia.

Inclusion criteria were participants (1) aged ≥18 years, (2) with the capacity to consent to take part in the VSGs, and (3) with access to a device and internet connection that would enable VSG participation.

#### Ethics Approval and Consent

The VSGs are part of the larger RDS Impact Project, conducted under the University College London Research Ethics Committee (8545/004: RDS Impact Study). See the study by Brotherhood et al [45] for details on the ethical procedures and consent.

#### Delivery

The RDS VSGs were conducted virtually using the GoToMeeting (GTM; LogMeIn Inc) videoconferencing platform. The group facilitators determined the number of sessions, with some groups held as one-off sessions, some as a series of 3 to 4 sessions, and other groups as ongoing. The format of the groups was also at the discretion of the facilitators, with some small groups being primarily experience-led and others being topic-focused or information-based.

#### Learnings From Field Testing

Initially, small online group discussions were offered as one-off, 1-hour-long information and experience-led sessions for RDS members. Sessions were subsequently increased to 1.5 hours to enable sufficient time for experience sharing alongside planned content. In addition, in the *independence and identity*, *grief and loss*, and *empowering your identity* groups, the facilitators felt that the connections made between group members and the scope of what could be covered within the group warranted a series of 4 sessions, rather than a one-off session. Once the 4 sessions were complete, participants were given the opportunity to continue to meet on a fortnightly or monthly basis, with light touch facilitation. Alongside these ongoing sessions, members who were connected with each other during the sessions were offered the option of one-to-one buddying. Further adaptations were made with regard to the timing of the sessions in the *empowering your identity* group for people living with dementia, which initially took place in the afternoon; however, the group members found it very difficult to concentrate at that time, so the facilitators moved the subsequent sessions to midmorning.

Facilitators also met as a group to provide feedback on challenges arising from VSG facilitation and strategies for managing them. Shared learnings from this discussion included the challenges of facilitating online groups, such as creating a safe and comfortable environment when not meeting face-to-face and assessing risk in a virtual context. There were a number of downsides to the virtual aspect of the groups, such as the anxiety of managing technological issues within the sessions, lack of in-person debriefing and reflection with colleagues, difficulty in trying to read attendees’ body language and nonverbal communication, and absence of boundaries between the facilitators’ work and home spaces. The benefits of using technology included the depth of conversations and insights shared by group members, indicating that they felt comfortable being open in the context and safety of their own homes, and the ability to privately address any of the individual participant’s concerns through the use of the chat function.

### Protocol for Round 3

#### Participants

In round 3, participation in small groups will be offered to the wider RDS membership, with an invitation window of 6 weeks. On the basis of the recruitment for rounds 1 and 2, we estimated 50 to 100 participants per round.

#### Group Size

In round 3, group size will be reduced from 12 to 10 members per group, in accordance with facilitator feedback and carer preferences in previous research [8].

#### Topics

The third round of small groups will include the topics from the first 2 rounds of groups, as well as a later stages program for carers and a creativity club for people living with dementia (Table 1).

#### Sampling Approach

All nonprofessional RDS members will receive an invitation via email to express their interest in the third round of small groups.

#### Data Handling

All VSGs, apart from the couples’ sessions in round 2 and the creativity club in round 3, will be recorded and automatically transcribed via the GTM platform. The recordings and transcriptions are stored securely on the University College London Data Safe Haven, which is only accessed by RDS Impact Study researchers. Once uploaded, the original files are deleted from GTM. As the accuracy of automated transcription is variable, meeting recordings will also be outsourced for professional transcription.

## Results

### Phase 3: Evaluation Plan

#### Overview

**Figure 2:**
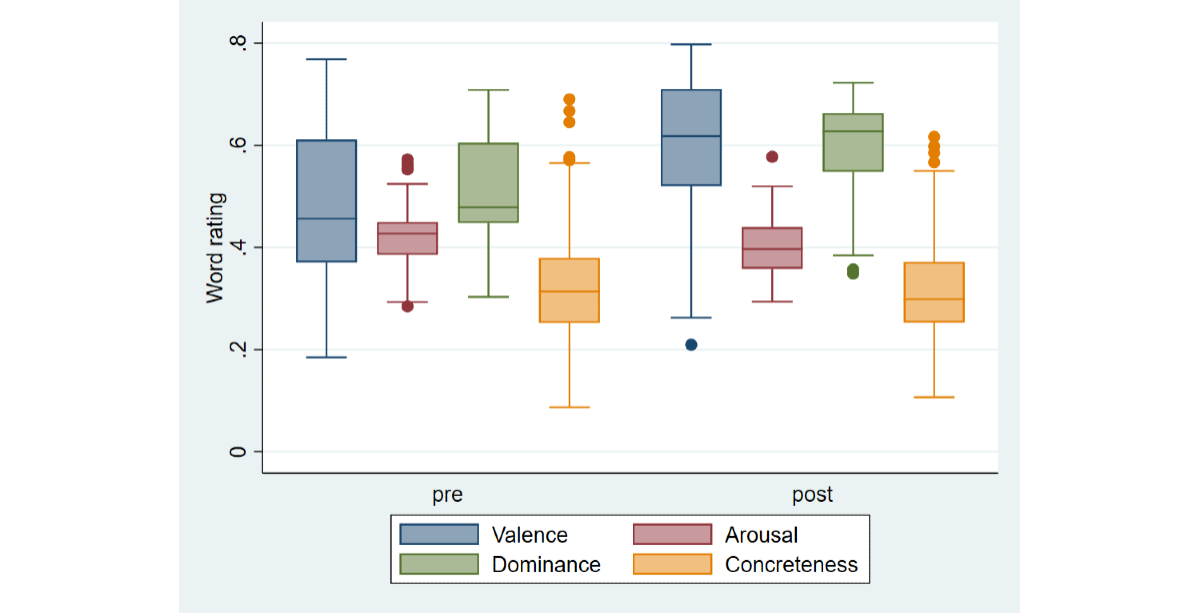
Pilot data from the “3 words” evaluation collected from participants (N=35) in wave 2 small-group conversations. Box and whisker plots show emotional valence, arousal, and dominance plus concreteness ratings of pre- and postsession words (N=301 words; 153 presession words and 148 postsession words). Linear mixed effects models were fitted for each linguistic score using STATA, including participant as a random effect (to account for nonindependence of words produced by each participant) and session theme as a fixed factor, and checking for normality of residuals (independent residual errors for the participants), heteroscedasticity, and linearity of the model. Pre-post differences were significant for all linguistic variables except concreteness, with valence and dominance scores increasing while arousal decreased (coefficients with *P* value, 95% CIs: valence coefficient=0.12, *P*<.001, 0.07 to −0.16; arousal coefficient=−0.02, *P*=.003, −0.04 to −0.01; dominance coefficient=0.08, *P*<.001, 0.04 to −0.11; concreteness coefficient=−0.013, *P*=.31, −0.04 to 0.01). A significantly different proportion of presession and postsession words were categorized by Linguistic Inquiry and Word Count as positive or negative emotion words (χ21=35.0, *P*<.001).

The first 2 rounds of VSGs were offered as a rapid service response to the pandemic, without a research plan to assess their impact. On the basis of field testing conducted during round 2 (Figure 2), a set of quantitative and qualitative investigations was designed to describe and measure the impact of round 3. Specific hypotheses for the quantitative investigations include (1) that session participation in small groups will be associated with increased in-the-moment well-being and (2) that participation, both within and across sessions, will be associated with enhanced social connectedness. Qualitative analysis will explore questions related to understanding how peer support groups work (eg, How are different types of social support delivered in peer support groups?) and specific questions related to the different themes of the groups (eg, In what ways are carers’ senses of identity impacted when supporting someone living with a rare dementia?).

#### Attendance, Demographics, and Geography

Participation in the groups will be evaluated against a range of factors, including gender, age, relationship to people living with dementia, diagnosis of person living with dementia, severity (judged by RDS facilitators using the Global Deterioration Scale) [46], frequency of RDS service use, and location. Key research questions include whether small online groups facilitate access to support for geographically dispersed members and later-stage carers, relative to standard face-to-face services typically delivered from our central London base.

#### Participant Ratings and Surveys

At the beginning and end of each session, participants will be asked to click a link to a web survey and (1) choose 3 words that describe how they are feeling in the moment and (2) complete the Canterbury Wellbeing Scale [47]. This will involve moving a web-based visual analog slider on a scale from 0 to 100 to indicate how they are feeling in the moment along the established 5 dimensions of happiness, wellness, interest, optimism, and confidence, including additional scales for stress and social connectedness. Three-word responses will be evaluated for emotional content using normative data for emotional valence, arousal, and dominance plus concreteness [48,49] and the Linguistic Inquiry and Word Count (LIWC) automated classification of positive and negative emotion words [50].

#### Linguistics

Online support groups also offer rich multimodal data on participants’ thoughts, emotions, and behaviors, which can deepen our understanding of support group processes. Voice recordings mean that linguistic analysis tools such as LIWC [50] have been used to explore the relationship between dropout rates and the level of emotional support received within sessions [51], and differences in the manner of expression between online and face-to-face support groups in young adults living with cancer [52].

The recorded conversations will be transcribed and evaluated cross-sectionally (within sessions) and longitudinally (across sessions) for evidence of thematic development and group cohesion. Specific features of interest include (1) participation, in terms of frequency, quantity, and equality of verbal contributions by individual participants and facilitators; (2) emotional content, such as the emotional valence, arousal and dominance of language used (quantified using norms in the study by Hollis and Westbury [48] and LIWC software, as per the “3 words analysis”); and (3) prevalence of specific features, such as incomplete propositions, hedges, signs of agreement (eg, in terms of use of names and grunts), and changes in pronoun (eg, “I” to “we”) and tense use (eg, past vs present vs future orientated utterances).

#### FaceReader

Facial video data have been analyzed with facial emotion recognition software such as FaceReader (version 7.0; Noldus Information Technology) to track changes in emotional regulation as markers of therapeutic effectiveness in individuals with borderline personality disorder [53]. Although not previously used with RDS groups, the exploratory use of these tools may yield novel metrics of group behavior, which through automation can be applied efficiently to future evaluations of the impact of online support groups.

Video recordings of the online meetings will be processed using FaceReader software, which classifies expressions into the categories of happy, sad, angry, surprised, scared, disgusted, and neutral and generates measures of the intensity of each individual emotion, valence (intensity of “happy” minus the intensity of the negative expression with the highest intensity), and arousal (based on the activation of 20 facial action units). In addition to quantifying overall differences in valence and arousal within and across sessions as the VSG conversations develop, FaceReader data will be used to explore (1) the relationship between the valence of facial emotion and verbal content of current conversation and (2) the cohesion of the group, taking the statistical variance of valence and arousal metrics among individual listeners to the current speaker in the group as proxies of cohesion.

#### Facilitator Ratings: Curative Climate Instrument

To complement participant ratings and observational linguistic and video data, facilitators of each group will be asked after each session to complete an adapted version of the Curative Climate Instrument [54] examining the processes of catharsis, cohesion, and insight within a small group. Originally designed as a measure for individual participants, facilitators will be asked to rate 13 of 14 statements reframed from a facilitator perspective (eg, “People were responsive to each other and made contact with each other through language, gesture, etc.”) for both levels of agreement (on a Likert scale from 1=strongly disagree to 7=strongly agree) and confidence in their agreement rating (from 1=extremely unconfident to 7=extremely confident).

### Phase 3: Qualitative Analysis Plan

The qualitative data (ie, transcriptions of the VSGs) will be analyzed to explore questions related to peer support groups overall, as well as questions that are specific to the different themes of each group, including those in the subsequent sections.

#### Qualitative Content Analysis

A directed content analysis [55] of all VSGs will be conducted to explore the question “How is social support delivered in peer support groups?,” with a coding framework based on the social support categories by Cutrona and Suhr [56] and the Social Support Behavior Code by Suhr et al [57]. Instrumental, tangible, emotional, and esteem support types will be coded for, and examples of each can be seen in Table 2.

**Table 2:** Qualitative content analysis.

Social support category	Example codes	Example data segments
Instrumental support	Suggestions and advice	“If you’re not sure about it (going to a day center), just go and have a look at what’s available...we were extremely reluctant and thought ‘Oh I don’t know’...We’d go through the activities and select what he wanted to do...That was quite helpful.”
Tangible support	Direct task	“I’m just going to put (helpful organization’s phone number) in the chat and if you (facilitator) could send it to people.”
Emotional support	Understanding and empathy	“The biggest problem I see is that we’ve all got the same problem that, unfortunately, we’re watching loved ones deteriorate. We know that there isn’t going to be any difference other than a slow deterioration, and we just adjust every time something happens.”
Esteem support	Compliments	“I think it is bureaucracy and you have done well to get through it and stand firm...I think you have been brilliant doing that.”

#### Thematic Analysis

Thematic analysis [58] of facilitator peer support sessions will explore the benefits and challenges of offering small peer support group discussions in a web-based format for people affected by rarer dementias to consolidate learning and develop recommendations for other facilitators embarking on similar initiatives. Benefits may relate to increased accessibility for those who would be unable to travel to in-person meetings because of their location, difficulties using public transport, or caring commitments. Challenges such as those relating to technology (eg, managing background noise and feedback), emotional impact (eg, lack of opportunities for informal conversations over coffee before and after meetings), and other factors will also be explored.

Thematic analysis will also be used to explore questions specific to the themes on which the small-group discussions were focused. For example, for the “Hope and dementia” group theme, how is the sense of hope challenged and sustained for people caring for a loved one with a diagnosis of a rare dementia? For the “independence and identity” group theme, how are the individual and shared identities of those living with a rare dementia and their carers impacted by the diagnosis?

### Phase 3

#### Economic Analysis

An exploratory analysis of the costs of developing the small online groups will be conducted using a microcosting approach from a societal perspective. We will microcost the development and delivery of the intervention to provide a clear representation of the costs of establishing these small groups.

Intervention costs will be requested from the groups. This list is not exhaustive but must include (1) cost of setting up the groups, (2) annual overheads, (3) cost of group materials (print costs and design costs), (4) salary costs for group and program facilitators, (5) training costs for facilitators, and (6) support costs for facilitators and volunteers.

We will also ask the groups to estimate any inputs, financial, time, or otherwise, so that these costs will also be accounted for.

#### Planned Analysis

We will take guidance from the UK Treasury Office Magenta Book in planning and designing the economic evaluation of this program [59].

This could include the following:

- Cost-benefit analysis using any participant questionnaire results to calculate quality-adjusted life years alongside the costs and benefits calculates the net present value of the program [60]. Cost-benefit analysis undertaken from a societal perspective allows the costs and benefits to be considered separately to consider a net monetary benefit or a ratio of benefits to costs, and considers all the costs and benefits to society [61]. Using deterministic sensitivity analysis, we will adjust the values for individual and multiple parameters and we will vary the discount rate from 0% to 3.5% to generate a range of scenarios, as recommended by the UK Treasury Green Book [62].
- Return on investment analysis, which would estimate, pound for pound, the return on investment from providing the VSGs.
- Cost consequence analysis works well with return on investment to quantify outcomes without traditional market values [61]. Cost consequence analysis allows for the outcomes to be quantified and related to costs for each separate course of action, where the final outcomes may be multidimensional; that is, to consider the range of relevant costs and outcomes, both anticipated and unanticipated.

The health economics component of this study will be written in accordance with the Consolidated Health Economic Evaluation Reporting Standards statement [63].

## Discussion

### Principal Predictions

We anticipate that connecting people affected by rare dementias together and providing a virtual space where they can share their experiences with others who are affected by the same conditions will be reflected by increased in-the-moment well-being outcomes, as well as an enhancement of social connectedness. We hope to develop a greater understanding of what works and does not in group peer support to improve service delivery for those affected by rare dementias.

To develop support tailored to the specific needs of people affected by rare dementias, it is vital that individuals with lived experience are involved in the design process. RDS members have played a significant role in the development of previous research projects [64,65], and their valuable input continues to shape both the RDS service and associated research, including the development of VSGs.

Although these groups were primarily developed in response to increased support needs during the COVID-19 pandemic, the recordings, pre- and postsession well-being measures, and participant feedback also provided an incredibly rich data source. We described a comprehensive evaluation plan using the data collected from these groups, the outcome of which will be used to further adapt and refine our web-based support provision. In addition, we hope that the learnings from these evaluations will be beneficial for other services that provide support for people with dementia as well as other health conditions, especially those that are rare and where individuals are geographically dispersed.

There are limitations with regard to this study, as the rapid setup of the groups meant that there was limited opportunity for comprehensive feedback and refinement of the group format and delivery ahead of the initial rounds. However, VSG development has also led to rapid learning for RDS staff regarding how to facilitate groups in a web-based context. The evaluation of these groups will further enable the development of a comprehensive framework for the delivery of online support for people affected by rare dementias across one-to-one, family, small-group, and large-group webinar formats.

In addition, because of the setup of the groups, all participants were required to familiarize themselves with the use of videoconferencing software, which likely would have excluded a number of individuals, particularly those living with dementia with additional accessibility needs. The study by O’Connell et al [66] suggests that participants should be provided with the option of joining via phone and video calls when conducting research remotely. This option was made available during the consenting process; however, it was not encouraged during the VSGs. If the participants had issues with internet connectivity during the session, the option of joining via phone was made available at that point. Future iterations might consider providing the option of joining via phone from the outset, rather than purely to overcome technical difficulties, although the impact on group dynamics of members joining via phone versus video may also need to be assessed.

### Conclusions and Future Directions

This paper has highlighted the importance of specific and targeted support delivered via VSG for people caring for or diagnosed with a rare dementia, the importance of coproduction, and the need for comprehensive evaluation of these groups to determine their effectiveness, and to further adapt and shape services to meet member needs in future. More broadly, the methods and findings of this work may also be of interest to other dementia-related service providers and providers of other long-term care conditions.

## Abbreviations

ESRC: Economic and Social Research Council
GTM: GoToMeeting
LIWC: Linguistic Inquiry and Word Count
RDS: Rare Dementia Support
VSG: videoconferencing support group
YOD: young-onset dementia
